# Structural model for ligand binding and channel opening of an insect gustatory receptor

**DOI:** 10.1016/j.jbc.2022.102573

**Published:** 2022-10-07

**Authors:** Satoshi Morinaga, Koji Nagata, Sayoko Ihara, Tomohiro Yumita, Yoshihito Niimura, Koji Sato, Kazushige Touhara

**Affiliations:** 1 Department of Applied Biological Chemistry, Graduate School of Agricultural and Life Sciences, The University of Tokyo, Tokyo, Japan; 2 International Research Center for Neurointelligence (WPI-IRCN), The University of Tokyo Institutes for Advanced Study, Tokyo, Japan

**Keywords:** silkworm, ion channel, receptor structure-function, fructose, homology modeling, docking, DSS, disuccinimidyl suberate, GR, gustatory receptor, meGFP, monomeric enhanced GFP, MTSET, 2-(trimethylammonium)ethyl methanethiosulfonate, bromide, OR, odorant receptor, RIPA, radio-immunoprecipitation assay, TIRF, total internal reflection fluorescence, TM, transmembrane

## Abstract

Insect gustatory receptors play roles in sensing tastants, such as sugars and bitter substances. We previously demonstrated that the BmGr9 silkworm gustatory receptor is a d-fructose–gated ion channel receptor. However, the molecular mechanism of how d-fructose could initiate channel opening were unclear. Herein, we present a structural model for a channel pore and a d-fructose–binding site in BmGr9. Since the membrane topology and oligomeric state of BmGr9 appeared to be similar to those of an insect odorant receptor coreceptor, Orco, we constructed a structural model of BmGr9 based on the cryo-EM Orco structure. Our site-directed mutagenesis data suggested that the transmembrane region 7 forms channel pore and controls channel gating. This model also suggested that a pocket formed by transmembrane helices 2 to 4 and 6 binds d-fructose. Using mutagenesis experiments in combination with docking simulations, we were able to determine the potent binding mode of d-fructose. Finally, based on these data, we propose a conformational change that leads to channel opening upon d-fructose binding. Taken together, these findings detail the molecular mechanism by which an insect gustatory receptor can be activated by its ligand molecule.

Animals utilize chemosensory systems to detect chemical information, such as odorants and taste substances, in their surrounding environment. In insects, the first step of chemical sensing is detection of chemical substances by chemosensory receptors expressed in olfactory and gustatory neurons. These receptors comprise odorant receptors (ORs) that bind odorants and pheromones in vapor and gustatory receptors (GRs) that recognize tastants such as sweet and bitter substances, as well as CO_2_ (1). In particular, the gustatory system detects nutritious and toxic compounds, and thus, it is crucial for food selection (2, 3).

Insect ORs and GRs comprise a multigene family with limited sequence homology, except for short sequences near the C-terminus (4, 5). ORs normally function as heteromers of an odorant-binding OR and the ubiquitous OR coreceptor, Orco, which is highly conserved among various insect species (6). Electrophysiological studies have demonstrated that OR-Orco heteromers function as a ligand-gated, nonselective cation channel (7, 8). This function was further supported by mutagenesis studies (9). Recently, Butterwick *et al*. (2018) and del Mármol *et al*. (2021) determined the cryo-EM structure of *Apocrypta bakeri* (Abak) Orco and *Machilis hrabei* OR5 (*Mh*OR5), revealing a tetrameric structure, reversal of the seven-transmembrane (TM) topology from that of a typical G protein–coupled receptor and a channel pore located at the center of the tetramer (10, 11).

It has been reported that *Drosophila melanogaster* Gr66a (DmGr66a) is involved in the detection of various taste substances, such as caffeine and l-canavanine, by coexpressing with other GRs, such as DmGr8a, Gr32a, and Gr93a (12, 13, 14, 15, 16, 17). However, unlike simple heteromeric OR complexes, combinatorial patterns of GRs have yet to be fully elucidated. One type of GR, a d-fructose receptor family termed the DmGr43a family, functions independent of other GRs. The d-fructose response was reproducibly recapitulated by expressing a single GR in heterologous expression systems such as *Xenopus laevis* oocytes and HEK293 cells (18). BmGr9 is a silkworm (*Bombyx mori*) GR belonging to the DmGr43a family. BmGr9 is narrowly tuned to d-fructose and appears to be a ligand-gated nonselective cation channel (18).

The present study aimed to address the molecular and structural bases underlying specific taste recognition in the insect gustatory system. A computational structural model for BmGr9 was constructed and used to identify a channel pore and d-fructose–binding domain in combination with site-directed mutagenesis. The three-dimensional (3D) AbakOrco structure was found to be a suitable reference for this purpose. The results provide structural insights into the ligand-induced conformational change that leads to channel opening in the insect GR.

## Results

### Oligomeric state and membrane topology of BmGr9

GR genes comprise the insect chemoreceptor superfamily together with OR genes (5). Previous studies for structural analysis of insect OR homomeric complexes reported that OR complexes are tetramers and each subunit forms seven transmembrane regions with N-terminus and C-terminus at the intracellular and extracellular sides, respectively (10, 11). We asked whether BmGr9 had a similar structure with ORs.

**Figure 1:**
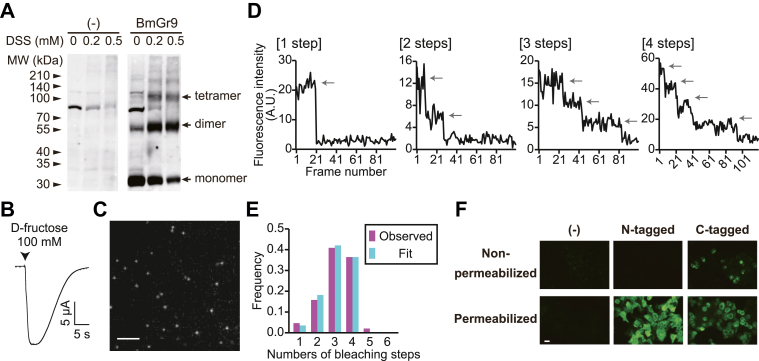
**Examination of the oligomeric state and the membrane topology of BmGr9.***A*, Western blotting of HEK293T cells transfected with BmGr9 using anti-BmGr9 antibody. Before Western blotting, HEK293T cells were treated with various concentrations of DSS shown above each lane to crosslink BmGr9 subunits in a functional complex. *B*, current response of a meGFP-BmGr9–expressing *Xenopus* oocyte responded to d-fructose. d-Fructose was applied at the timing indicated by arrowhead for 3 s. *C*, actual fluorescence image of oocyte membrane expressing meGFP-BmGr9. The scale bar represents 2 μm. *D*, representative time course of fluorescence intensity at single fluorescence spot. Fluorescence intensity was photobleached in a stepwise manner at the timing indicated by *arrows*. *E*, frequency of bleaching step numbers. Observed frequency was well fitted with the binomial distribution, assuming that BmGr9 forms a tetramer and the probability that meGFP fluorescent was 0.776. n = 569. *F*, antibody staining of HEK293T cells transfected with none (−) or BmGr9 fused with *myc*-tag at the N-terminus or C-terminus. Anti-myc staining was performed under nonpermeabilized or permeabilized conditions. The scale bar represents 20 μm. DSS, disuccinimidyl suberate; meGFP, monomeric enhanced GFP.

First, the oligomeric state of BmGr9 was examined. We treated HEK293T cells expressing BmGr9 with disuccinimidyl suberate to introduce a crosslink within the functional BmGr9. Western blotting with the anti-BmGr9 antibody revealed the presence of three major bands at ∼30, ∼55, and ∼100 kDa that were not observed in nontransfected cells (Fig. 1*A*). This result suggested that ∼30, ∼55, and ∼100 kDa likely correspond to monomers, dimers, and tetramers of BmGr9, respectively. Thus, BmGr9 appears to exist as a homo-oligomer.

To verify the oligomeric state in an environment closer to the actual physiological state, single-molecule imaging of GFP-tagged BmGr9 expressed in *Xenopus* oocytes was performed using Total Internal Reflection Fluorescence (TIRF) microscopy. BmGr9 was fused with monomeric enhanced GFP (meGFP) at the N-terminus (meGFP-BmGr9). The meGFP-BmGr9 responded to d-fructose in *Xenopus* oocytes and was evident as numerous fluorescent spots on the cell surface under a TIRF microscope (Fig. 1, *B* and *C*). The fluorescence of each spot was photobleached in a stepwise manner (Fig. 1*D*). Of the tetramer, pentamer, and hexamer models, only the binomial distribution of tetramers fits well with the observed frequency (Fig. 1*E*). Together with the above biochemical data, BmGr9 appears to function as a homotetramer.

To determine the membrane topology, BmGr9 genes were fused with the myc-tag at the N- or C-terminus, and immunofluorescence staining of transfected HEK293T cells was performed using anti-myc antibody under a permeabilized or nonpermeabilized condition (Fig. 1*F*). The findings suggest that the N-terminus of BmGr9 is positioned at the intracellular side and the C-terminus at the extracellular side. This topology appears to be the same as that of insect ORs and is also consistent with previous studies on silkworm GRs (19).

### Construction of 3D structural model of BmGr9

Since BmGr9 appears to have the same oligomeric composition with insect homomeric ORs, we performed a homology modeling of BmGr9 by using the cryo-EM structure of AbakOrco as a template (10).

**Figure 2:**
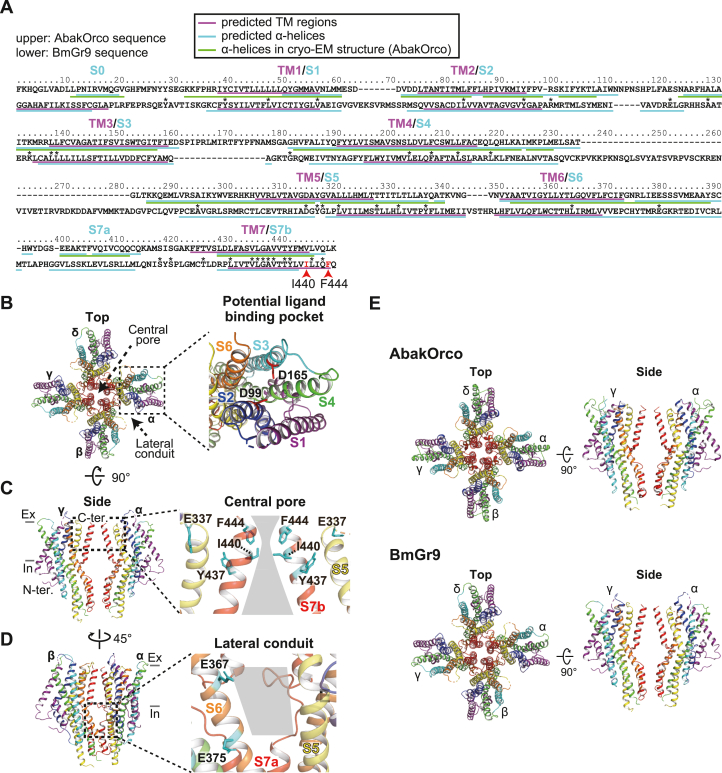
**BmGr9 structural model based on Orco cryo-EM structure.***A*, the pairwise alignment of BmGr9 and AbakOrco using AlignMe. *Magenta* underlines show the predicted TM regions, *cyan* underlines show the predicted helix structures, and *green* underlines show the helix regions of the cryo-EM structure of AbakOrco. *B*, 3D structural model of BmGr9 observed from the *top*. BmGr9 forms a tetramer and each subunit is named α-δ. In the enlarged inset, side chains of D99 and D165 residues are shown by *red sticks*. *C*, observation from the side. For clarity, subunits β and δ are omitted. In the enlarged inset, the structure of the outer pore region is shown. Side chains of E337, Y437, and I440 residues are shown by *cyan sticks*. Possible ion conduction pathway is highlighted by a *gray background*. *D*, observation from the direction rotated 45° at the vertical axis from (*C*). For clarity, subunits γ and δ are omitted. In the enlarged inset, the structure of the lateral conduit is shown. Side chains of E367 and E375 are shown by *cyan sticks*. *E*, comparison of cryo-EM structure of AbakOrco and 3D structural model of BmGr9. Each structure is viewed from the extracellular side or the membrane plane. TM, transmembrane.

Pairwise sequence alignments of AbakOrco and BmGr9 were established using various alignment programs (Multalin, T-Coffee, Clustal Omega, AlignMe, and TM-Aligner). The reliability of the alignments was determined based on the positional consistency of the predicted TM regions between Orco and BmGr9. The best alignment was obtained with AlignMe (Fig. 2*A*). Next, a 3D-model of BmGr9 was constructed using MODELLER based on this alignment (Fig. 2, *B*–*E*). In this model, four subunits are lined with four-fold symmetry, forming a tetrameric structure, and the channel pore near the extracellular side is positioned at the center of the tetramer (Fig. 2, *B* and *C*). Each subunit has seven membrane-spanning helical segments (S1–S7). S1 to S4 and S6 form an open pocket toward the extracellular side. Previous studies on insect ORs have implicated that the corresponding pocket functions as the ligand-binding site. Thus, hereafter, we refer to this region as the potential ligand-binding pocket (Fig. 2*B*). The central pore at the extracellular side is connected to the sideways located between two adjacent subunits termed lateral conduits on the intracellular side, which may play a role as an ion-conduction pathway (Fig. 2*D*) (10).

### Surveying amino acid residues important for ionotropic receptor function

**Figure 3:**
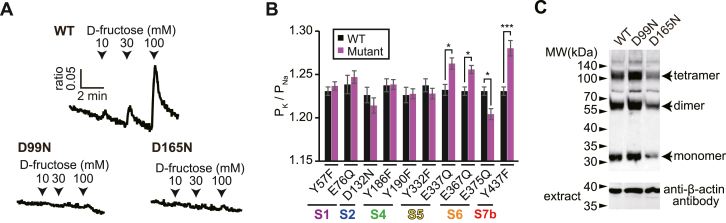
**Surveying amino acid residues important for the ionotropic receptor function**. *A*, Ca^2+^ response of HEK293T cells transfected with WT BmGr9 or each mutant. d-Fructose was applied at the timing indicated by arrowheads for 15 s. *B*, permeability ratio of K^+^ ions to Na^+^ ions of functional BmGr9 mutants. The values of each mutant were compared to those of the WT measured on the same day. *Black bars* indicate the values of the WT, and *magenta bars* indicate the values of mutants shown at the *bottom*. Mean ± SEM; n = 10 to 17; unpaired Student’s *t* test; Bonferroni correction; ∗*p* < 0.05; ∗∗∗*p* < 0.001. *C*, expression level of D99N and D165N mutants on plasma membrane. *Arrows* indicate the bands corresponding to monomers, dimers, or tetramers.

To validate the molecular model, point mutations were introduced at the amino acid residues predicted to be involved in the channel function. We previously showed that aspartic acid (D), glutamic acid (E), and tyrosine (Y) residues located around TM5–TM7 regions of ORs contribute to the ion selectivity (D299 in TM5 and E356 in TM6 of BmOr1, Y464 in TM7 of BmOrco) (9). To examine whether these findings are applicable to BmGr9, the three amino acid residues conserved within the four BmGr9 orthologous genes were mutated (Fig. S1*A*). Ten of the twelve mutants exhibited a variable d-fructose response, whereas the two D99N and D165N mutants were dead (Figs. S1, *B*, *C*, and 3*A*). The permeability ratios of K^+^ ions to Na^+^ ions (P_K_/P_Na_) were measured in the 10 functional mutants in an ion substitution experiment. The ratios of E337Q, E367Q, E375Q, and Y437F were significantly different from those of the WT (Figs. 3*B* and S1, *D*–*E*). On the BmGr9 model, E337 and Y437 do not directly face to the possible ion-conduction pathway (Fig. 2*C*). However, they are located near regions potentially controlling the channel gating of BmGr9 (described later in Fig. 5). In addition, E367 and E375 are facing to the possible ion-conduction pathway in the lateral conduit.

On the other hand, the two nonvalid mutants, D99N and D165N, protrude toward the inside of the pocket (Fig. 2*B*). To examine whether the two mutants were properly expressed on the cell surface, we biotinylated the proteins expressed on the plasma membrane of HEK293T cells transfected with WT BmGr9 or D99N or D165N mutant and purified them with avidin-beads. Western blotting with anti-BmGr9 antibody clearly revealed bands corresponding to the size of monomer, dimer, and tetramer in each mutant lane (Fig. 3*C*). The findings suggested that D99N and D165N were properly translocated to the cell surface, and that the loss-of-function is due to an effect on ligand-binding and/or channel gating. Overall, these results support the structural accuracy of the ion-conduction pathway and the ligand-binding site of the BmGr9 model.

### Targeting residues located at the channel gate

**Figure 4:**
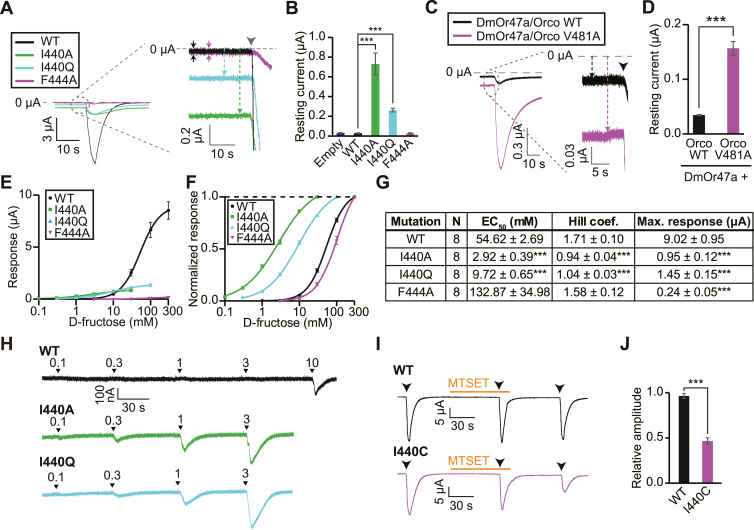
**Targeting residues located at the channel gate.***A*, superimposed response waveforms of *Xenopus* oocytes injected with WT BmGr9 or I440 or F444 mutants (*lower left*) and its partially enlarged image (*right*). Each oocyte was clamped at -80 mV and 10 mM (I440A), 30 mM (I440Q), or 100 mM (WT, F444A). d-fructose was applied at the timing indicated by an arrowhead for 3 s to confirm the functional expression of receptors. The amplitudes of resting current are shown by *arrows*. *B*, the amplitudes of the resting current. n = 5. *C*, representative waveforms of oocytes injected with WT or mutant cRNA of DmOrco together with DmOr47a. Oocytes were clamped at -80 mV. VUAA1, an agonist of Orco, was applied at a concentration of 100 μM at the timing indicated by an arrowhead for 3 s. *D*, resting current of WT DmOrco or V481A mutant together with DmOr47a. n = 5. *E*, dose-response of WT BmGr9 and I440A, I440Q, and F444A mutants. *F*, normalized dose-response curves. The response amplitude of d-fructose at a concentration of 30 mM (I440A), 100 mM (I440Q), or 300 mM (WT, F444A) was set to one at each measurement. *G*, the EC_50_ values, Hill coefficient, and max response of normalized response. *H*, representative response waveforms of WT BmGr9 and I440 mutants toward low concentrations of d-fructose. d-Fructose was applied at the timing indicated by arrowheads for 3 s. *I*, effect of MTSET treatment on WT BmGr9 and I440C mutant. Oocytes injected with WT BmGr9 or I440C mutant were stimulated three times with 100 mM d-fructose at the timing indicated by arrowheads for 3 s and treated with MTSET before and after the second d-fructose stimulation. *J*, response induced by d-fructose before and after MTSET treatment. Relative amplitude is the ratio of the d-fructose current after MTSET treatment to that before treatment. Mean ± SEM; unpaired Student’s *t* test; Bonferroni correction in (*B* and *G*); ∗∗∗*p* < 0.001. MTSET, 2-(trimethylammonium)ethyl methanethiosulfonate, bromide.

A few mutations in the hydrophobic residues at the extracellular side of AbakOrco S7b increase ligand sensitivity, suggesting that these residues contribute to the control of channel gating (10). Thus, we introduced mutations at I440 and F444, which correspond to the hydrophobic residues of AbakOrco and which protrude toward the pore lumen in the model (Fig. 2, *A* and *C*), to examine receptor channel activity. Interestingly, I440 mutants showed an increase in an inward current (resting current) compared to that of the WT at a holding potential of −80 mV (Fig. 4, *A* and *B*), which was considered to be caused by an increased cation influx through the channel without ligand stimulation. A similar increase in resting current has been observed for some ligand-gated ion channels upon introduction of a mutation in a conserved hydrophobic residue building the pore module (20, 21, 22).

To confirm that an increase in the resting current of I440A mutant-injected oocytes was due to a spontaneous inward current, the I440A-injected oocytes in the resting state were treated with ruthenium red that inhibits the cation flux through BmGr9 (18). The inhibition ratio of the resting current in the oocytes injected with I440A was on the same extent with that for the d-fructose response of the WT (Fig. S2, *A* and *B*); therefore, the increased resting current was suggested to be derived from an increase in the basal ion conduction through BmGr9. In addition, we confirmed that the membrane expression level of I440A was comparable to that of the WT (Fig. S2*C*), showing an increase in the resting current was not due to an elevated expression level of I440A. To examine whether a corresponding mutation in Orco could increase the resting current, we introduced an alanine mutation at V481 in DmOrco. As expected, the V481A mutant coexpressed with a ligand-binding subunit, DmOr47a, showed an increased resting current compared to that of the WT (Fig. 4, *C* and *D*).

While I440 mutants showed decreased responsiveness to d-fructose compared to the WT, the EC_50_ and the threshold of d-fructose were shifted toward the lower concentration side (Fig. 4, *E*–*H*). These changes in d-fructose sensitivity suggested that the mutations of BmGr9 I440 generate a larger ion-conduction pathway and/or destabilize the closed conformation similar to the effect of the corresponding AbakOrco mutation (10). In contrast, the F444A mutation impaired d-fructose responsiveness more drastically than I440 mutations (Fig. 4, *E*–*G*), which could be due to a shift of equilibrium relation between open and closed conformation, decrease in single-channel conductance, or decrease in the expression level. Furthermore, we used a Cys-modifying reagent, 2-(trimethylammonium)ethyl methanethiosulfonate, bromide (MTSET), to confirm that I440 is positioned at the pore lumen. MTSET reacts irreversibly with Cys residues, which adds a positively charged group to the side chain of Cys, resulting in cation flux inhibition. MTSET application did not cause a change in d-fructose responsiveness of oocytes expressing WT BmGr9, whereas the response of I440C was markedly inhibited by MTSET application (Fig. 4 , *I*and *J*). These results indicate that the hydrophobic residues on S7b, including I440, constitute the channel-pore module and contribute to channel gating of BmGr9.

### Targeting residues involved in regulation of channel gating

**Figure 5:**
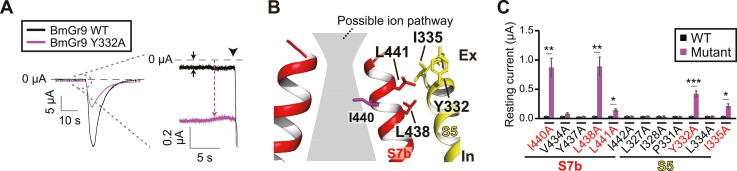
**Targeting residues involved in regulation of channel gating**. *A*, representative waveforms of oocytes injected with WT or Y332A cRNA of BmGr9. Oocytes were clamped at −80 mV. d-Fructose was applied at a concentration of 100 mM at the timing indicated by an arrowhead for 3 s. *B*, the positions of residues whose mutations increased the resting current amplitude in the 3D-model. *C*, Resting current amplitude of each mutant. Mean ± SEM; n = 5; unpaired Student’s *t* test; Bonferroni correction; ∗*p* < 0.05; ∗∗*p* < 0.01; ∗∗∗*p* < 0.001.

Since the point mutation on I440 resulted in an increase in the resting current, the residues around I440 seemed to be involved in channel gating. Additionally, during the course of mutagenesis experiments of conserved amino acid residues in Figure 3, we incidentally found that an alanine mutation at Y332 induced an increase in the resting current (Fig. 5*A*). Y332 is located at the interface between S5 and S7b and the vicinity of I440 in the model (Fig. 5*B*). In typical voltage-gated channels, channel gating is controlled by the helix adjacent to the pore-forming helix and some mutations of residues located between these helices cause the channels to be constitutively active (23, 24). In BmGr9, S5 is the only helix that contacts S7b in the model. Therefore, we speculated that a point mutation of a residue located at the interface between S5 and S7b caused BmGr9 to become constitutively active, resulting in an increased resting current. We selected 11 residues located at the interface between S5 and S7b, six residues from S5 (L327, I328, P331, Y332, L334, and I335), and five residues from S7b (V434, Y437, L438, L441, and I442). An alanine mutation was introduced at these residues. All 11 mutants responded to d-fructose, verifying their functional expression. Mutations at Y332, I335, L438, and L441 resulted in a significant increase in the resting current compared to that of the WT (Fig. 5*C*). In addition, the Y332F mutation increased d-fructose responsiveness (Fig. S1, *B* and *C*), which might have been due to the disturbance of gating. These affected residues are all located near the I440 residue (Fig. 5*B*) and are likely involved in the control of channel gating.

### Targeting residues located within the potential ligand-binding pocket

**Figure 6:**
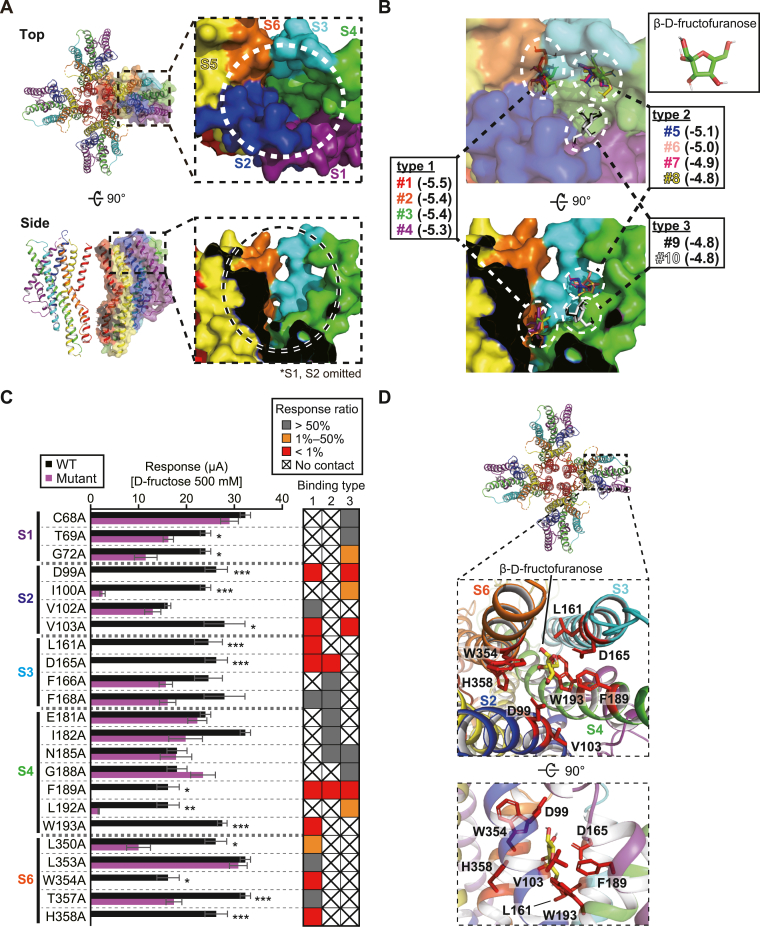
**Targeting amino acid residues located within a potential ligand-binding pocket.***A*, surface representation of the BmGr9 structural model. The model is viewed from the *top* (*upper*) or side (*lower*). In the *left panels*, the translucent surface model is superimposed on a single subunit of the cartoon model. In the *right* enlargements, the position of the putative ligand-binding pocket is enclosed by *dashed circles*. S1 and S2 helices are omitted in the *lower* enlarged image for clarity. *B*, 10 energetically stable modes of β-d-fructofuranose in the flexible-docking simulation (numbered #1–#10 in the order of the energy stabilities). The structure of β-d-fructofuranose is shown in the *upper right*. The model is viewed from the top (*upper*) or side (*lower*). β-d-fructofuranose positions are clearly classified into types 1 to 3. Numbers in parentheses followed by each mode show the affinity (kcal/mol). *C*, response amplitude of BmGr9 mutants toward 3 s stimulation of 500 mM d-fructose. In the *right*, contact residue candidates in binding types 1 to 3 are color-coded by the relative response amplitude compared to the WT (*gray*: >50%, *orange*: 1%–50%, *red*: <1%). Mean ± SEM; n = 3 to 6; unpaired Student’s *t* test; Bonferroni correction; ∗*p* < 0.05; ∗∗*p* < 0.01; ∗∗∗*p* < 0.001. *D*, the positions of the important residues for d-fructose responsiveness. In the enlarged image, the *red stick* model shows side chains of residues whose alanine mutations eliminated d-fructose responsiveness. The position of β-d-fructofuranose molecule is that of binding mode #2 belonging to type 1.

The BmGr9 model indicates that there is an open pocket formed by S1–S4 and S6 at the outer side of each subunit that may allow the entry of d-fructose into the pocket from the extracellular side. Docking simulations of BmGr9 and d-fructose were performed using AutoDock Vina. β-d-Fructofuranose was selected as an isomeric form of d-fructose because d-fructose binds to proteins in this form in about 80% of d-fructose–protein complex structures deposited in the Protein Data Bank. In the putative ligand-binding pocket, energetically favorable 10 binding modes were chosen that could clearly be classified into three types according to the binding positions of β-d-fructofuranose (type 1: #1–4, type 2: #5–8, type 3: #9, 10) (Fig. 6, *A* and *B*). Next, we identified contact residues with β-d-fructofuranose in each binding mode using the PDBePISA program to explore macromolecular interfaces (25) (Table S1). An alanine mutation was introduced at each residue included in the table and the d-fructose responsiveness of each mutant was assessed. D99A, V103A (on S2), L161A, D165A (on S3), F189A, W193A (on S4), W354A, and H358A (on S6) almost completely lost the d-fructose responsiveness (Fig. 6*C*). The results suggested that the eight residues are likely located in the β-d-fructofuranose–binding site. Since all these residues were included in the type 1 binding site of β-d-fructofuranose (Fig. 6*D*), we conclude that the type 1 binding site represents the most probable d-fructose–binding site on BmGr9.

The cryo-EM structure of *Mh*OR5 in complex with its ligand, eugenol, was recently reported (11). To compare the ligand-binding modes in BmGr9 and *Mh*OR5, BmGr9 and *Mh*OR5 were superimposed and the alignment of the positionally corresponding amino acid residues was constructed. Comparison of the amino acid residues important for ligand responses in BmGr9 and *Mh*OR5 revealed that these residues fairly corresponded to each other (Fig. S3, *A* and *B*). Although physicochemical characteristics of these corresponding amino-acid residues were different between each other, this result suggests the importance of spatial arrangement of amino acid residues in the 3D structure of a chemosensory receptor for the ligand binding.

## Discussion

**Figure 7:**
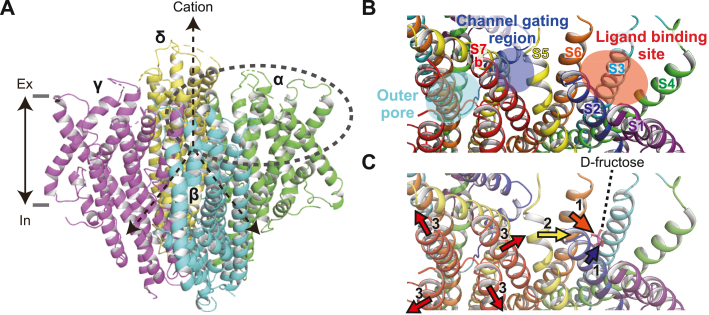
**A model of conformational changes leading to channel opening upon ligand binding.***A*, a 3D-structural model of a homotetrameric BmGr9 complex. Each subunit is colored separately. *B*, close-up around one subunit near the extracellular side (*gray dashed circle* in *A*). Outer pore is constituted from S7b of four subunits. Residues located at the interface between S5 and S7b were suggested to be related to channel gating. d-fructose was suggested to bind to a pocket formed by S2, S3, S4, and S6. They are located within the putative ligand-binding pocket on the model. *C*, a hypothetical structural movement toward an open conformation of BmGr9. Upon binding of d-fructose, S2 and S6 are pulled toward d-fructose (step 1), then S5 moves to the opposite side of the pore in coordination with S2 and S6 (step 2), and eventually S7b moves to the opposite side of the pore in coordination with S5 (step 3), resulting in opening of an outer pore.

In this study, we performed homology modeling of the BmGr9 d-fructose–binding silkworm GR based on the cryo-EM structure of the Orco, an OR coreceptor (10). The model was highly consistent with the site-directed mutagenesis results. There is little amino acid sequence homology between the insect OR and GR families, for example, the amino acid sequence identity between BmGr9 and *B. mori* Orco is 12.5%, and these families have been considered distantly related genes from the perspective of partial sequence homology around the C-terminus and conserved exon-intron structure (26, 27). Although it remains to be determined whether the current model can be applied to other GRs and while the 3D structure remains to be determined by crystallization or cryo-EM, our biochemical and modeling results suggest that the insect OR and GR families are functionally and structurally related more closely than expected at the amino acid sequence level. We demonstrated a structural model for an ion channel pore and a d-fructose–binding site and proposed a molecular mechanism by which a conformational change upon ligand binding leads to channel opening (Fig. 7).

A mutation at I440 in BmGr9, which is directed toward the lumen side of a putative channel pore in the model, resulted in a constitutive conductive phenotype and altered ligand sensitivity (Fig. 4, *A*, *B*, and *E*–*H*). The same constitutive channel opening was observed upon introduction of a mutation at the corresponding residue in Orco (Fig. 4, *C* and *D*). Furthermore, modification of I440 with the MTSET reagent after cysteine mutation led to a decrease in d-fructose responsiveness (Fig. 4, *I* and *J*). These results suggest that I440 protrudes the inner side of the pore and likely constitutes a hydrophobic gate in a manner similar to various ion channel families (28, 29, 30, 31). Consistently, a stretch of nine amino acid residues encompassing I440 near the C-terminus (TYhhhhhQF, T436–F444 of BmGr9; in many cases, h is a hydrophobic amino acid) is highly conserved within the insect GRs and is assumed to be a motif sequence (Fig. S4) (26), confirming the critical contribution toward the channel function of this site. Among amino acid residues affecting the ion selectivity of BmGr9, E337, Y437, and I440 were located around the outer pore region in the model, while E367 and E375 were located on the intracellular side. Butterwick *et al*. reported the presence of lateral conduits in Orco formed at the interfaces between adjacent subunits (S5–S7) that were proposed to function as intracellular ion pathways (10). In our model, the E367 and E375 residues in BmGr9 are located around the region corresponding to the lateral conduits (Fig. 2*D*). In addition, our ConSurf analysis revealed that the amino acid locus of E375, Y437, and I440 show evolutionarily conservative tendency among GRs (Fig. S4, *A* and *B*), indicating that these residues play generally important roles for GR function, such as the formation of the ion pathway. The collective data support the proposal that in the predicted channel pore formed by S7, I440 is involved in the entry of cations into the pore, and E367 and E375 help pull down the cation into the cytosol.

In previous studies on insect ORs, it was considered that the ligands bound to the pocket located at the outer side of the tetrameric structure (32, 33, 34, 35). Thus, we focused on the pocket and successfully identified eight candidate residues forming a ligand-binding site in the pocket (Fig. 6). These residues show the evolutionarily conservative tendency among BmGr9 orthologous genes, implying that these residues have functions related to a ligand binding (Fig. S4, *A* and *B*). The ligand affinities and specificities of noncovalent carbohydrate-binding proteins are substantially influenced by hydrogen bonds and CH-π interactions (36, 37, 38, 39). Hydrogen bonds are mainly formed between the hydroxyl groups of carbohydrates and polar residues, while CH-π interactions are formed between the C and H bonds on carbohydrate rings and aromatic residues. A statistical survey revealed that, of polar residues, aspartic acid and asparagine are favored within the close vicinity of carbohydrates. In addition, three aromatic residues—histidine, tryptophan, and tyrosine—also frequently occur near carbohydrates (39). The eight candidates of d-fructose–binding residues of BmGr9 (Fig. 6*D*) included five of the aforementioned residues (D99, D165, W193, W354, and H358), which supports our ligand-binding site model.

There were only two polar residues (D99 and D165) in the putative d-fructose–binding site of BmGr9. In contrast, in high-affinity protein–carbohydrate complexes, such as the periplasmic sugar receptors (K_d_ ≈ 10^−6^–10^−7^ M), almost all the hydroxyl groups of a carbohydrate form a complicated hydrogen-bond network with multiple polar residues (36). Therefore, the formation of only a few hydrogen bonds with d-fructose might tune the sensitivity of BmGr9 to the appropriate range (mM) for taste perception. Although the detailed binding mode of d-fructose could not be examined with this model, further analysis of the intermolecular interaction between BmGr9 and d-fructose by high-resolution structural solution of the BmGr9–d-fructose complex will clarify the molecular basis for the ligand affinities and specificities.

The increased resting current caused by the mutations of residues located at the interface between S5 and S7b suggests that channel gating is controlled by the interaction of S5 and S7b. The potential ligand-binding pocket is away from the channel pore and consists of five helices other than S5 and S7b. Therefore, a conformational change wave is required to be transmitted from the potential ligand-binding pocket to S7b through S5. Among the helices that interact with d-fructose, S2 and S6 contact S5 on the extracellular side, indicating that the movement of S5 is likely controlled by S2 and/or S6. Thus, the following model for conformational change leading to channel opening is conceivable. When d-fructose binds to the potential ligand-binding pocket, the relative positions of S2, S3, S4, and S6 change to a new state. The movement of S2 and S6 is transmitted to S5, followed by S7b, leading to the opening of the channel gate (Fig. 7*C*). Among the type 1 binding modes, a hydrogen bond is formed between D99 on S2 and β-d-fructofuranose in mode #2, which could be a motive force for channel opening (Fig. 6*B* and Table S1). In addition, the D99 locus is conserved among 97.5% of BmGr9 orthologous genes (Fig. S4*B*), implying that this residue plays an important role in the conformational change upon d-fructose binding.

In olfactory receptor neurons, ligand-specific ORs and Orco expressed in a large population of ORNs form a heterocomplex that functions as a ligand-gated channel (7, 9, 40, 41). Similarly, coexpression of multiple GRs is required for the response to various substances in GR neurons (13, 15, 16, 17, 42, 43). In addition, some GRs, such as DmGr66a, are expressed in a broad range of GR neurons and are involved in the response to various tastants. Thus, these GRs may function as coreceptors for ligand-specific GRs in a fashion similar to Orco (12, 17, 44). The combinatorial pattern, however, seems to be more complicated than that for ORs and has not been fully understood. Our findings suggest that GRs function as tetramers as with ORs, giving an insight into how GRs constitute functional complexes *in vivo*.

Recently, the cryo-EM structure of an insect OR-odorant complex was revealed (11). In the comparison of ligand-binding sites in BmGr9 and *Mh*OR5, the structurally corresponding amino acid residues appear to contribute to the ligand recognition (Fig. S3, *A* and *B*). Further, del Mármol *et al*. also pointed out the contribution of π-stacking interaction in the *Mh*OR5-eugenol binding in the light of an importance of aromatic residues. In addition, they suggested that a conformational change occurred upon the ligand binding was transmitted to the central pore *via* S5, which is consistent with our hypothesis about the conformational change of BmGr9. These findings support the validity of our BmGr9 model and suggest that molecular mechanisms underlying the ligand-binding and subsequent conformational changes in ORs and GRs may be evolutionarily conserved.

We also constructed a BmGr9 model using AlphaFold2, which was a recently developed approach for predicting protein structures from primary sequences (45). The sequence of monomeric BmGr9 was entered in AlphaFold2 because the amino-acid number of a BmGr9 tetramer (1796 residues) exceeds a limit for AlphaFold2 prediction. The MODELLER model and the AlphaFold2 model have similar structures in the membrane topology and the arrangement of the helix regions (Fig. S5*A*). In addition, a possible ligand-binding pocket is also constituted in the AlphaFold2 model (Fig. S5*B*).

In summary, this study provides a structural model that consistently explains the ligand-gated channel function of GRs. Although the detailed 3D structures of insect GRs have to be elucidated, the knowledge obtained in this study should contribute to an increased understanding of the mechanism by which insect GRs form a complex oligomer to sense and discriminate various taste substances.

## Experimental procedures

### Reagents

d-Fructose was purchased from Wako, VUAA1 was purchased from Vitas-M Laboratory, Ruthenium Red was purchased from NACALAI TESQUE INC., and MTSET was purchased from Toronto Research Chemicals.

### Preparation of anti-BmGr9 antibody

Rabbit antiserum raised against a synthetized peptide encoding the sequence of amino acids 1 to 14 of BmGr9 [NH_2_-C-MPPSPDLRADEPKT-COOH ] (Eurofins Genomics) was affinity-purified using the SulfoLink Immobilization Kit for Peptides (Thermo Fisher Scientific).

### Cell culture and transfection

HEK293T cells were grown in low glucose Dulbecco's modified Eagle's medium (DMEM) containing L-glutamine and phenol red (Wako Chemicals) supplemented with 10% fetal bovine serum and maintained at 37 °C in a humidified atmosphere containing 5% CO_2_. Cells were passaged when they reached approximately 80% confluence. For Western blotting, immunocytochemistry, and calcium imaging, 4 μg of pME18S vector for BmGr9 was transfected into HEK293T cells seeded in a 35 mm glass-bottomed dish (Iwaki) precoated with poly-D-lysine (Sigma-Aldrich) using 10 μl Lipofectamine 2000 (Invitrogen).

### Detection of BmGr9 proteins using anti-BmGr9 antibody

#### Detection of oligomers using a crosslinking agent

At 40 to 44 h after transfection, HEK293T cells were incubated with the solution containing the crosslinking agent disuccinimidyl suberate (Thermo Fisher Scientific) at 37 °C for 15 min. The reaction was stopped by adding Tris–HCl (pH 7.5) to a final concentration of 50 mM, and cells were lysed by sonication in 0.1% radio-immunoprecipitation assay (RIPA) buffer (1% Triton X-100, 0.1% SDS, 0.1% sodium deoxycholate, 10 mM Tris-HCl (pH7.5), 150 mM NaCl, and 5 mM EDTA). After centrifugation, the supernatant was subjected to SDS-PAGE with 9% Tris-Glycine/SDS-gel and 5 M urea. Proteins on the gel were transferred to a polyvinylidene fluoride membrane by the wet method using transfer buffer (15 mM Tris, 100 mM glycine, 10% methanol) at 4 °C and 100 V for 2 h. The membrane was shaken in stripping buffer (100 mM 2-mercaptoethanol, 2% SDS, 62.5 mM Tris HCl, pH 6.7) at 55 °C for 15 min to enhance sensitivity in the detection of a membrane protein (46). After washing with 0.1% Tween in Tris buffered saline, the membrane was blocked by incubation with 5% skim milk overnight. The membrane was incubated with anti-BmGr9 antibody and then with anti-rabbit-horseradish peroxidase secondary antibody (Biosource) for 1 h at room temperature. Signals were visualized by chemiluminescence using ImmunoStar LD (Wako) and detected using ImageQuant LAS4000 mini (GE Healthcare).

#### Detection of BmGr9 proteins expressed on plasma membrane

After 40 to 44 h transfection, proteins expressed on the plasma membrane were biotinylated using EZ-Link-NHS-Biotin (Thermo Fisher Scientific) at 4 °C for 30 min, then quenched by Tris–HCl (pH 7.5, final concentration 50 mM). The cells were pelleted by centrifugation, washed with PBS, and lysed by sonication in 0.1% RIPA buffer. After centrifugation, the supernatant was subjected to a binding reaction with NeutrAvidin beads (Thermo Fisher Scientific) at 4 °C for 2 h. After washing avidin-beads with 0.1% RIPA buffer, bound proteins were eluted with Laemmli sample buffer supplemented with urea to a final concentration of 5 M. The sample was subjected to SDS-PAGE, followed by Western blotting as described above.

### Immunocytochemistry

At 40 to 44 h after transfection, HEK293T cells expressing myc-tagged BmGr9 were subjected to immunostaining for BmGr9. For permeabilization, cells were fixed with 4% paraformaldehyde in PBS for 10 min and then permeabilized with 0.1% Triton X100 in PBS for 5 min. After washing with PBS, the cells were incubated with anti-myc antibody (9E10; Santa Cruz Biotechnology), washed, incubated with anti-mouse IgG Alexa Fluor 488 conjugated secondary antibody (Thermo Fisher Scientific). For nonpermeabilization, cells were incubated with anti-myc antibody in staining solution (DMEM supplemented with 5% fetal bovine serum and 1 M Hepes (pH 7.4) to produce a final concentration of 10 mM) on ice for 1 h. After washing the cells with Ringer’s solution (140 mM NaCl, 5.6 mM KCl, 2.0 mM MgCl_2_, 2.0 mM CaCl_2_, 1.25 mM KH_2_PO_4_, 9.4 mM glucose, 2.0 mM sodium pyruvate, and 5 mM Hepes; pH 7.4), cells were further incubated with the secondary antibody for 1 h, followed by washing with PBS and fixation with 1% paraformaldehyde in PBS. Samples were observed by a fluorescence microscopy using a model IX73 microscope (Olympus).

### Calcium imaging

Calcium imaging of HEK293T cells was performed as previously described (18). At 40 to 48 h after transfection, HEK293T cells were loaded with 2.5 μM Fura-2 AM (Thermo Fisher Scientific) at 37 °C for 20 min. After washing the cells with Ringer’s solution, the ligand in Ringer’s solution was applied with a peristaltic pump for 15 s at a flow rate of 1.5 ml/min. Data collection and analysis were performed using Aquacosmos (Hamamatsu Photonics K.K.).

### Single-molecule imaging using a TIRF microscopy

Stage V to VII *Xenopus* oocytes were treated with 2 mg/ml of collagenase B (Roche Diagnostics) in Ca^2+^-free saline solution (82.5 mM NaCl, 2 mM KCl, 1 mM MgCl2, and 5 mM Hepes; pH 7.5) for 2 h at 18 °C, injected with 250 pg cRNA of meGFP-BmGr9, incubated for 3 days, and observed. To remove the vitelline membrane surrounding the plasma membrane just before observation, the oocytes were incubated in 2.5× Barth’s solution containing 1 mg/ml hyaluronidase (Sigma-Aldrich) and 1 unit/ml neuraminidase (Sigma-Aldrich) for 15 min at room temperature (47). After shrinkage of the plasma membrane due to high osmolality, the vitelline membrane was carefully removed with tweezers under a stereomicroscope. The oocytes were then placed on a 35 mm glass-bottomed dish (Iwaki) and subjected to observation with IX83/TIRF (Olympus) using a UAPON 100XOTIRF objective lens (oil immersion, NA 1.49). The power of the laser beam used for excitation was 10 mW. Fluorescence images were sequentially recorded with an iXon Ultra (ANDOR) at 113 ms/frame. Image analysis was performed using MetaMorph software (Molecular Devices). The bleaching step at each spot was determined by visual inspection. Theoretical step distributions of tetramer, pentamer, and hexamer models were calculated by applying the binomial distribution with changing the probability that meGFP is fluorescent (described as *P* in Fig. 1*E*). Testing whether the observed distribution fit to the theoretical distributions above by the chi-squared test rejected all but the tetramer at the 5% significance level within the *p*-value range of 0.759 to 0.791.

### *In silico* prediction of membrane topology and secondary structures

The TM regions and helical structures were predicted using the web servers TMHMM server v. 2.0 (http://www.cbs.dtu.dk/services/TMHMM/) and PredictProtein (https://www.predictprotein.org/), respectively (48, 49). The predicted helical structures up to four residues, which correspond to one helical turn, were omitted.

### Construction of the BmGr9 structural model using MODELLER

The cryo-EM structure of AbakOrco complex (Protein Data Bank accession number 6C70) (10) was used as the template structure. Pairwise alignments of Orco and BmGr9 were obtained using the following programs.

Multalin (http://multalin.toulouse.inra.fr/multalin/)

T-Coffee (http://tcoffee.vitalit.ch/apps/tcoffee/do:regular)

Clustal Omega (http://www.ebi.ac.uk/Tools/msa/clustalo/)

AlignMe (http://www.bioinfo.mpg.de/AlignMe/AlignMe.html)

TM-Aligner (http://lms.snu.edu.in/TMAligner/index.php)

The reliability of each pairwise alignment was assessed by the positional consistency of the predicted TM regions between AbakOrco and BmGr9, which indicated that the pairwise alignment obtained with AlignMe was the most reliable. The molecular model of BmGr9 was built using MODELLER (https://salilab.org/modeller/) (50) based on the pairwise alignment obtained using AlignMe. Symmetry restraints were applied for C^α^ atoms between any chain pairs to obtain a homotetrameric structure with the same subunit conformation. All molecular graphics were created using open-source PyMOL (https://github.com/schrodinger/pymol-open-source).

### Construction of the BmGr9 structural model using AlphaFold2

The primary sequence of BmGr9 was deposited in the webserver easy to use the version of AlphaFold2 (https://colab.research.google.com/drive/1LVPSOf4L502F21RWBmYJJYYLDlOU2NTL) (45).

### Comparison of the protein structure models

The protein structures (the BmGr9 model and *Mh*OR5 in Fig. S3 and the BmGr9 models constructed using MODELLER and AlphaFold2 in Fig. S5) were compared using the DALI server (http://ekhidna2.biocenter.helsinki.fi/dali/) (51).

### Multiple sequence alignment and evolutionary conservation of amino acid residues on BmGr9

Multiple sequence alignment of BmGr9 orthologous genes was obtained using MAFFT-7.245 (52). The sequences for analyzing GR consensus sequence were collected from four distantly related insects, *B. mori* (76 GRs), *D. melanogaster* (68 GRs), *Dendroctonus ponderosae* (60 GRs), and *Cephus cinctus* (36 GRs) (27, 53, 54, 55). The sequences for analyzing BmGr9 consensus sequence were collected using the protein BLAST server (https://blast.ncbi.nlm.nih.gov/Blast.cgi) with entering BmGr9 sequence as query. Only one sequence from one insect genus was selected in the order of BLAST score with a lower bound on GPRGr25, whose BLAST score was the lowest within four kinds of d-fructose receptors. Multiple sequence alignments of 240 sequences for GR consensus and 122 sequences for BmGr9 ortholog consensus are constructed using MAFFT and the alignments are entered in the ConSurf server (https://consurf.tau.ac.il/consurf_index.php) (56) to analyze the consensus sequences.

### Site-directed mutagenesis

Point mutations were introduced by PCR using a reaction mixture containing PrimeSTAR Max DNA polymerase buffer (TaKaRa Bio), 10 pg of template cDNA, and 0.33 μM oligonucleotide primers. The sequences of the mutants were determined by Fasmac (Kanagawa).

### Functional analysis of receptors in *Xenopus* oocytes

cRNA was synthesized from a linearized modified pSPUTK vector. Oocytes treated with collagenase were microinjected with 0.5 to 12.5 ng of WT BmGr9 or each mutant cRNA or 6.25 ng of DmOr47a together with 6.25 ng of WT DmOrco or its V481A mutant. Injected oocytes were incubated for 3 to 5 days at 18 °C in Barth’s solution (88 mM NaCl, 1 mM KCl, 0.3 mM Ca(NO_3_)_2_, 0.4 mM CaCl_2_, 0.8 mM MgSO_4_, 2.4 mM NaHCO_3_, and 15 mM Hepes; pH 7.6) supplemented with 84.7 mg/ml gentamicin. Whole-cell currents were recorded using a two-electrode voltage clamp technique. Intracellular glass electrodes were filled with 3 M KCl. Signals were amplified with an OC-725C amplifier (Warner Instruments), low-pass filtered at 50 Hz and digitized at 1 kHz. The theoretical basal resting current was measured using a model cell circuit of OC-725C. The control bath solution contained 115 mM NaCl, 2.5 mM KCl, 1.8 mM MgSO_4_, 2.4 mM NaHCO_3_, and 10 mM Hepes (pH 7.2). All sugars were directly diluted into the bath solution and VUAA1 was first prepared in dimethyl sulfoxide at 100 mM as stock solution, which was then added to the stock solution at 0.1% to give a final concentration of 100 μM. Stimulant solutions were delivered *via* a silicon tube connected to computer-driven solenoid valves and the stimulation duration was 3 s in the response measurement experiments. In the measurements of current-voltage relationship, clamping voltage was shifted from −25 mV to +20 mV by applying ramp pulse for 0.65 s after 5 seconds from the onset of d-fructose stimulation. Start timing of voltage-shift was set after 5 s from the onset of d-fructose. In the experiment using ruthenium red, 100 μM ruthenium red was prepared in the same way as 100 μM VUAA1 described above. In the measurement of a d-fructose response, ruthenium red was applied for 60 s together with d-fructose immediately after 10 s stimulation of 100 mM d-fructose alone. In the measurement of the resting current, ruthenium red was applied for 60 s. In the experiment using MTSET, each oocyte was treated with MTSET (2.5 mM) for 73 s (60 s before, 3 s during, and 10 s after d-fructose application). In ion substitution experiments, the following solutions were used: 98 mM XCl, 1 mM MgCl_2_, 5 mM Hepes, titrated to pH 7.4 with XOH (X = Na^+^ or K^+^). To calculate the permeability ratio, the following extended form of the Goldman-Hodgkin-Katz flux equation was used (9):

$$\frac{Pk}{PNa}=\frac{{\left[Na^{+}\right]}_{o}}{{\left[K^{+}\right]}_{o}\times \exp\left(\frac{\Delta Erev\times F}{RT}\right)}$$

$$\left(\Delta Erev=Erev\left(Na^{+}\right)-Erev\left(K^{+}\right)\right)$$

The junction potential was corrected for the measurement of E_rev_. Data acquisition and analysis were carried out using Digidata1322A (Axon Instruments), pCLAMP software (Axon Instruments), and Igor pro (WaveMetrics).

### Docking simulation

The atomic coordinates of β-d-fructofuranose were acquired from the Protein Data Bank under the ligand ID FRU. Molecular docking was performed using AutoDock Vina (57). The search space for molecular docking was manually set around the potential ligand-binding pocket of the BmGr9 model (see Fig. 6*A*). Protein-ligand interfaces in the docking modes were analyzed using PDBePISA (https://www.ebi.ac.uk/pdbe/pisa/) (25).

## Data availability

All data will be available upon request.

## Supporting information

This article contains supporting information (11).

## Conflict of interest

The authors declare that they have no conflicts of interest with the contents of this article.
