# Supplementary material for: Structural model for ligand binding and channel opening of an insect gustatory receptor
Source: J Biol Chem. 2022 Oct 7;298(11):102573. doi: 10.1016/j.jbc.2022.102573 (PMC9643425; doi:10.1016/j.jbc.2022.102573)
Supplement: Supporting information [file mmc1.pdf]

Supporting information

Structural model for ligand binding and channel opening of an insect gustatory receptor

Authors

Satoshi Morinaga,[1] Koji Nagata,[1] Sayoko Ihara,[1] Tomohiro Yumita,[1] Yoshihito Niimura,[1,3] Koji Sato,[1] and Kazushige Touhara[1,2]

[1]Department of Applied Biological Chemistry, Graduate School of Agricultural and Life Sciences, The University of Tokyo, Tokyo 113-8657, Japan
[2]International Research Center for Neurointelligence (WPI-IRCN), The University of Tokyo Institutes for Advanced Study, Tokyo 113-0033, Japan
[3]Present Address: Department of Veterinary Sciences, Faculty of Agriculture, University of Miyazaki, Miyazaki 889-2192, Japan

Correspondence should be addressed to Sayoko Ihara at asihara@mail.ecc.u-tokyo.ac.jp or Kazushige Touhara at ktouhara@mail.ecc.u-tokyo.ac.jp.

This article contains supplementary Figures S1, S2, S3, S4, S5, and Table S1.

A

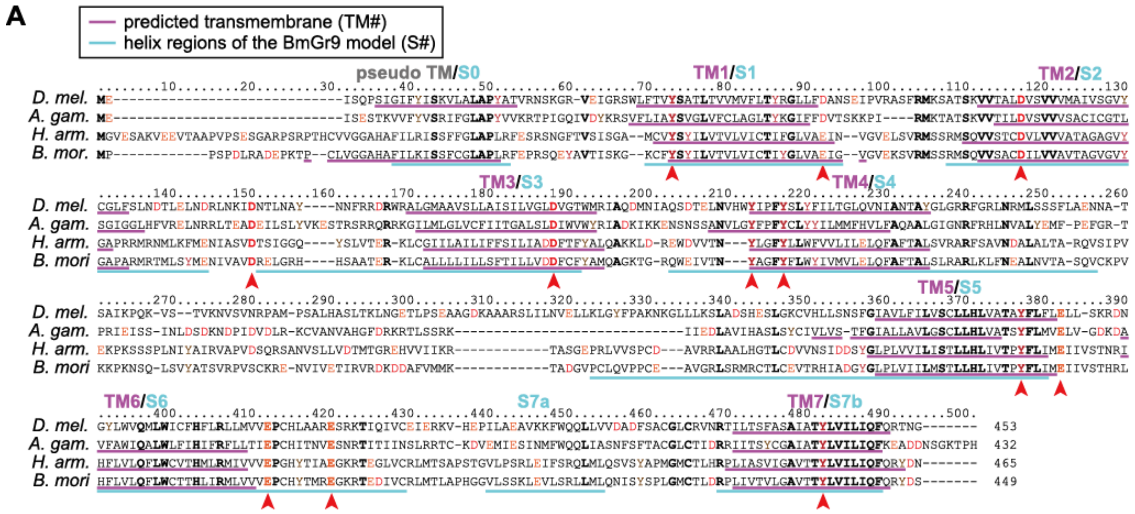

B

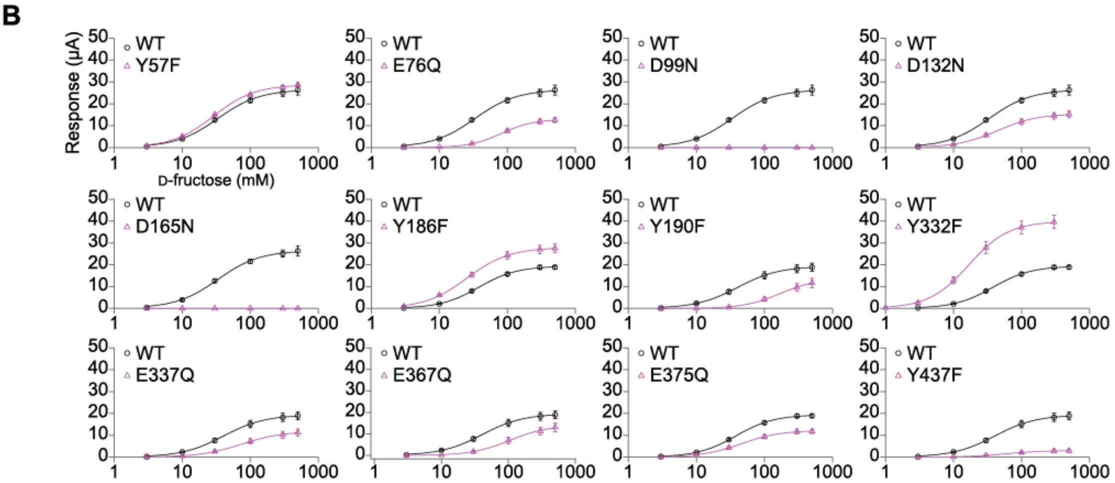

C

Mutation	N	EC_{50} (mM)	Hill coef.	Max response (μA)
WT	6	33.94 ± 3.55	1.45 ± 0.11	26.74 ± 2.57
Y57F	6	28.80 ± 2.59	1.45 ± 0.04	28.73 ± 1.13
E76Q	6	82.71 ± 3.68***	1.78 ± 0.10	13.04 ± 1.30**
D99N	5	-	-	-
D132N	6	45.03 ± 2.91	1.48 ± 0.03	15.52 ± 1.82*
D165N	5	-	-	-

Mutation	N	EC_{50} (mM)	Hill coef.	Max response (μA)
WT	6	38.26 ± 1.93	1.56 ± 0.04	19.38 ± 0.90
Y186F	6	25.70 ± 1.71	1.41 ± 0.02*	27.87 ± 2.21*
Y332F	6	17.25 ± 1.32	1.49 ± 0.01	39.89 ± 3.26***
E375Q	6	46.68 ± 2.80	1.56 ± 0.08	12.20 ± 1.19**

Mutation	N	EC_{50} (mM)	Hill coef.	Max response (μA)
WT	6	42.58 ± 3.72	1.42 ± 0.09	19.38 ± 1.97
Y190F	6	182.41 ± 18.61***	1.68 ± 0.10	13.81 ± 2.85
E337Q	6	82.49 ± 16.32	1.44 ± 0.06	12.03 ± 1.88
E367Q	6	116.18 ± 18.93*	1.58 ± 0.09	14.33 ± 2.56
Y437F	5	74.30 ± 18.07	1.52 ± 0.07	3.03 ± 0.83***

D

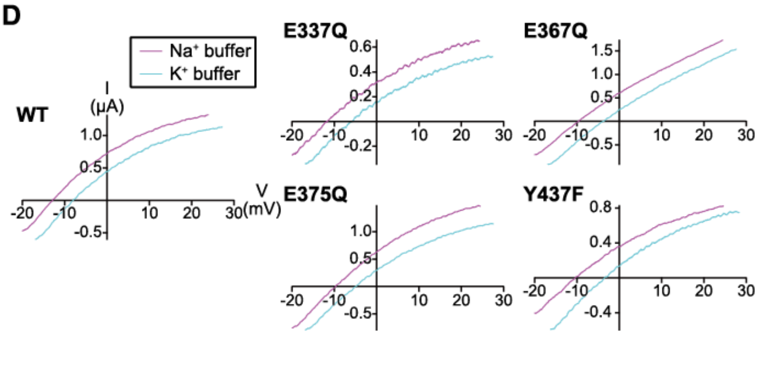

E

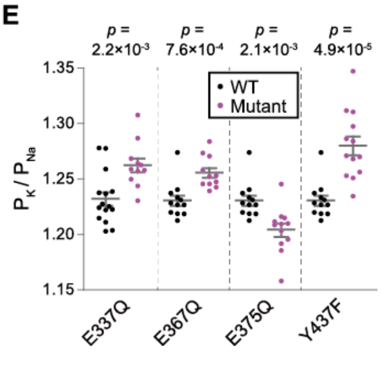

Figure S1. Mutational effect of highly conserved amino acid residues.

(A) Multiple sequence alignment of BmGr9 orthologous genes. Magenta underlines show the predicted TM regions and cyan underlines show the helix regions of the BmGr9 model. Red arrowheads show the Asp, Glu, and Tyr residues conserved within all genes. *D. mel.*, *Drosophila melanogaster*; *A. gam.*, *Anopheles gambiae*; *H. arm.*, *Helicoverpa armigera*; *B. mor.*, *Bombyx mori*. (B) Dose response curves of BmGr9 mutants. Each oocyte was injected with 1 ng cRNA of BmGr9 mutant. Mean ± SEM. (C) EC_{50} values, Hill coefficient, and max response of each mutant. Each table shows the result in one day. Mean ± SEM; unpaired Student's *t*-test (vs. the WT); Bonferroni correction; *, $p<0.05$; **, $p<0.01$; ***, $p<0.001$. (D) Representative I-V curves of WT BmGr9 and mutants showing significantly altered ion permeability ratios in Na^+ buffer (magenta) or K^+ buffer (cyan) with the same oocyte. (E) Dot plots of P_K/P_{Na} with each single oocyte. Mean ± SEM values are shown together as bars.

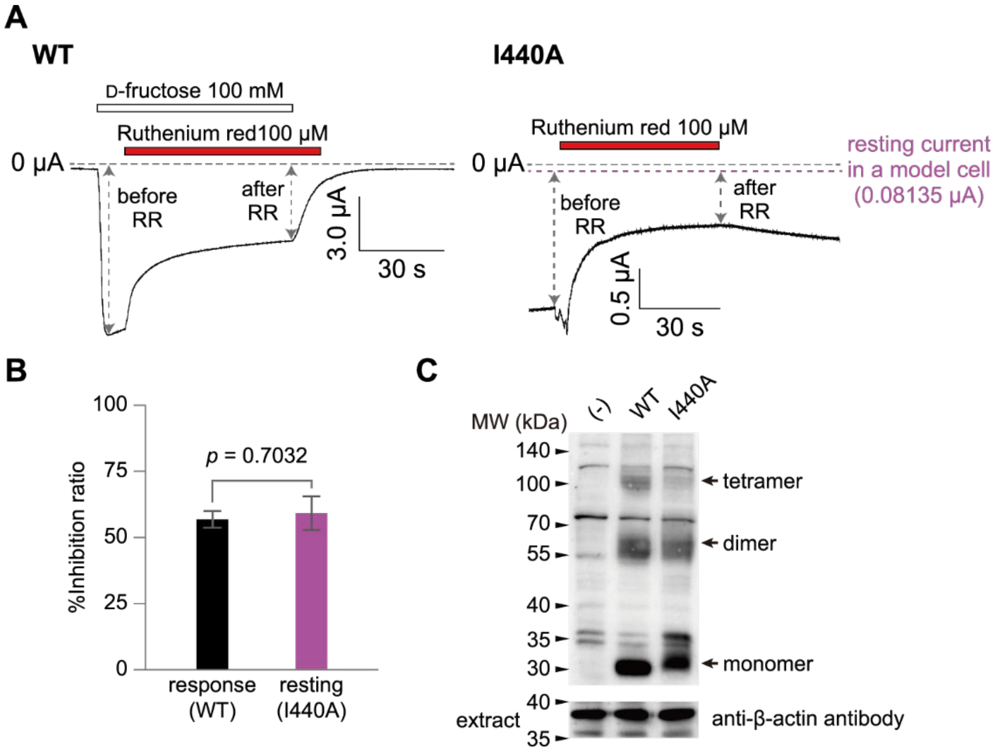

Figure S2. Examination of the cause of the increased resting current by I440A mutation. (A) Effect of ruthenium red on the D-fructose response of the WT and the resting current of the oocytes injected with I440A. (B) Inhibition ratios. Mean ± SEM; n = 7 (WT) or 5 (I440A). (C) Expression level of the WT and the I440A mutant on plasma membrane. Arrows indicate the bands corresponding to monomers, dimers, or tetramers.

A

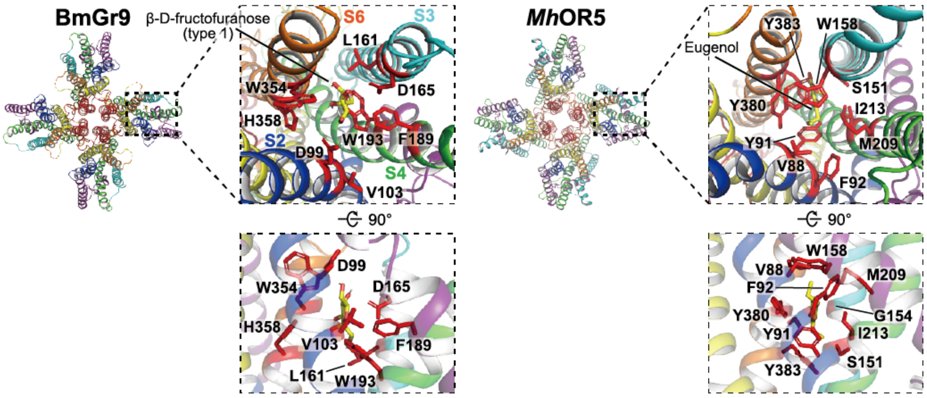

B

	Helix	BmGr9 MODELLER position	BmGr9 MODELLER Ala mutation	*Mh*OR5 position	*Mh*OR5 Ala mutation
Positionally corresponding residues	S2	D99	Significant effect on ligand response	V88	Significant effect on ligand response
		L102	Not significant	Y91	Significant effect on ligand response
		V103	Significant effect on ligand response	F92	Significant effect on ligand response
	S3	F158	No contact	S151	Significant effect on ligand response
		L161	Significant effect on ligand response	G154	Significant effect on ligand response
		D165	Significant effect on ligand response	W158	Significant effect on ligand response
	S4	F189	Significant effect on ligand response	M209	Significant effect on ligand response
		Y193	Significant effect on ligand response	I213	Significant effect on ligand response
	S6	W354	Significant effect on ligand response	Y380	Significant effect on ligand response
		T357	Significant effect on ligand response	Y383	Significant effect on ligand response
		H358	Significant effect on ligand response	I384	No contact

Legend: Significant effect on ligand response (magenta); Not significant (cyan); No contact (crossed box)

Figure S3. A comparison of the ligand-binding sites of BmGr9 and *Mh*OR5. (A) The ligand-binding site structures of BmGr9 and *Mh*OR5. The amino acid residues that appear to have important contribution to the ligand binding are represented by red sticks. (B) Structurally corresponding residues of BmGr9 and *Mh*OR5 and their mutational effect on a ligand response (see Fig. 6C and del Mármol et al., 2021).

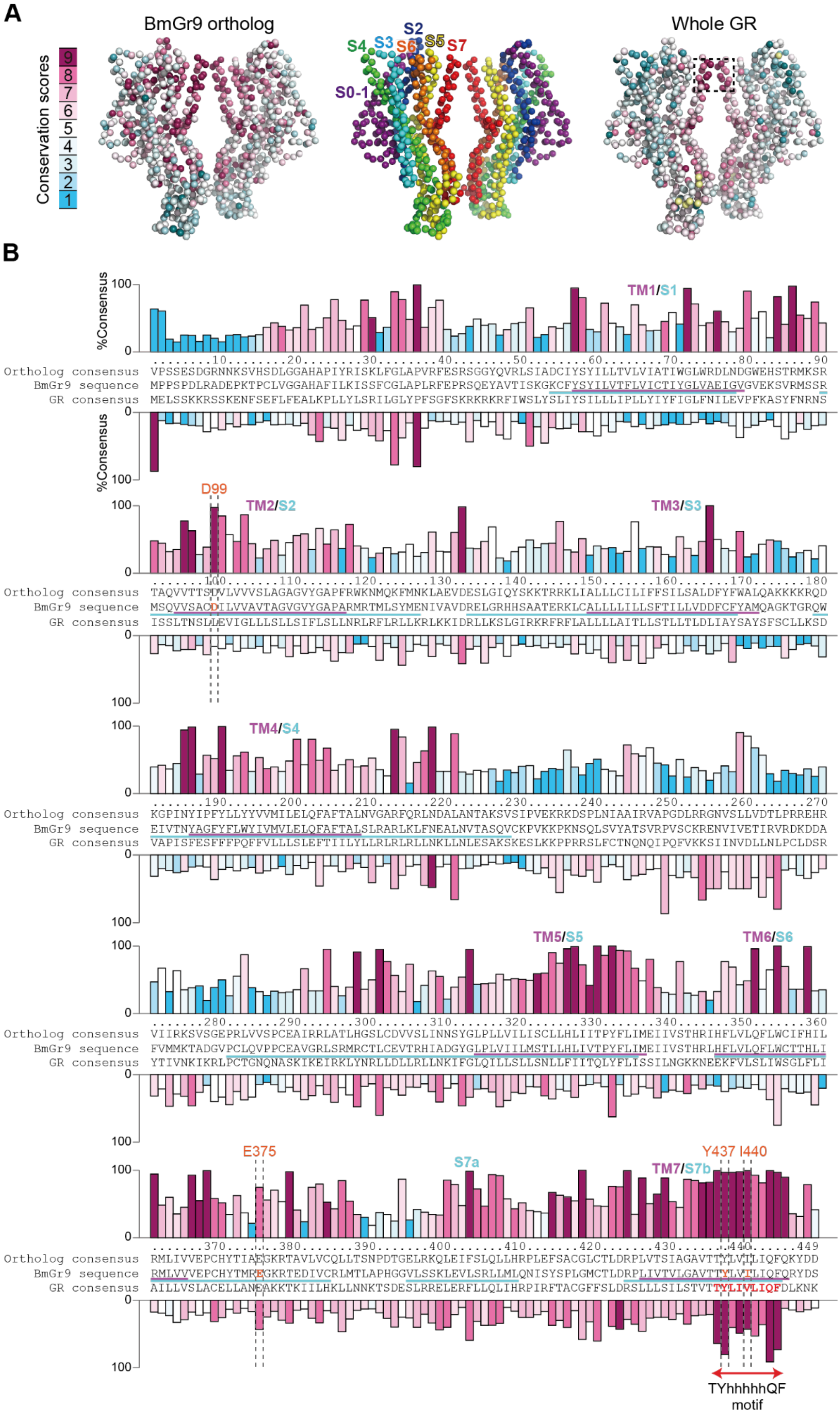
A
BmGr9 ortholog
Whole GR
Conservation scores
S0-1
S2
S3
S4
S5
S6
S7
B
%Consensus
Ortholog consensus
BmGr9 sequence
GR consensus
TM1/S1
TM2/S2
TM3/S3
TM4/S4
TM5/S5
TM6/S6
TM7/S7b
S7a
D99
E375
Y437
I440
TYhhhhhQF motif

Figure S4. Evolutionary conservation of amino acid residues in BmGr9. (A) ConSurf conservation scores of amino acid residues in BmGr9 among BmGr9 orthologous genes (left) and whole GRs (right). The scores are aligned to the Cα atoms of the BmGr9 model. The position of the TYhhhhhQF motif (T436–F444 of BmGr9) is shown by a dotted-line square in (A) and a red two-headed arrow in (B). (B) The identities of consensus sequences among BmGr9 orthologous genes (upper) and whole GRs (lower) are plotted. Bar height, the percent identities; bar color, ConSurf conservation scores.

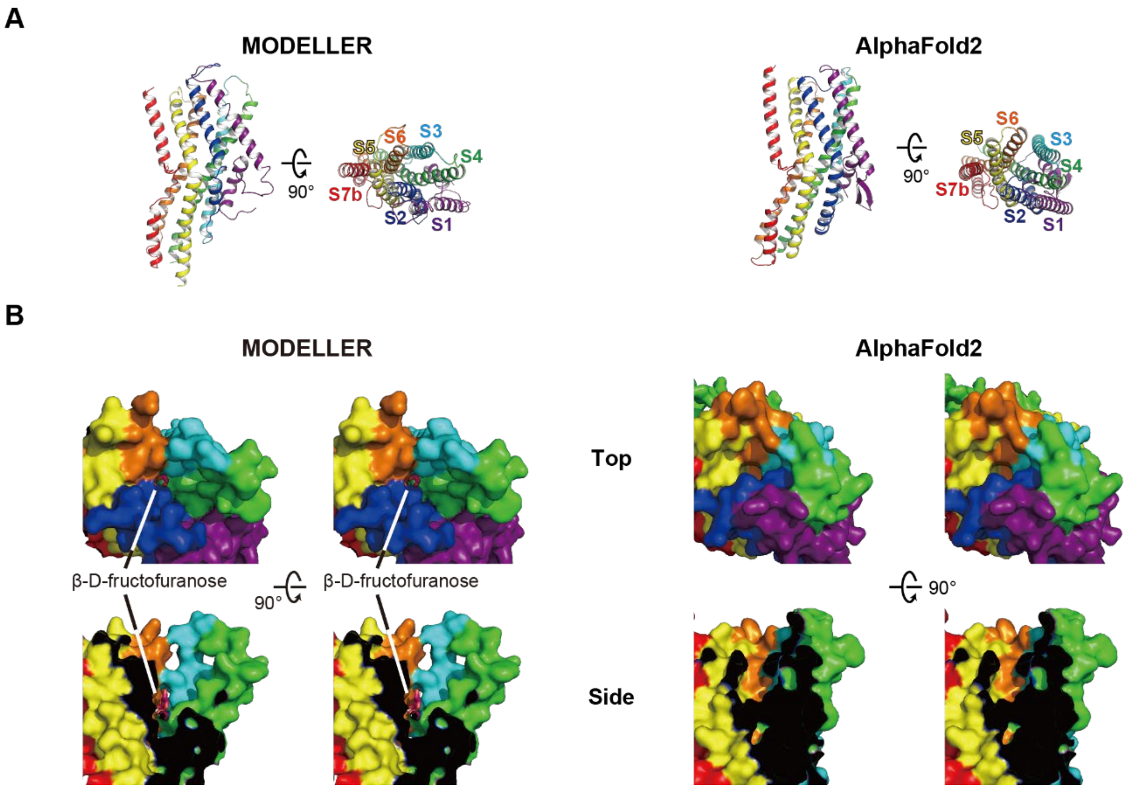

Figure S5. Construction of a BmGr9 structural model using AlphaFold2. (A) The MODELLER model (left) and the AlphaFold2 model (right). The RMSD value between the two models excluding the intracellular loop regions of 52 amino acid residues between S4 and S5 is 3.6 Å. (B) Stereoviews showing the surface representation of the putative ligand-binding sites in the MODELLER model (left) and the AlphaFold2 model (right). The position of β-D-fructofuranose molecule is that of binding mode #2 belonging to type 1. In the side views, S1 and S2 helices are omitted for clarity.

Mode [type]	Res.	Helix	Bond	ASA	BSA	ΔG^i
#1 [1]	D99	S2		65.4	1.5	-0.02
	V102			17.0	6.0	0.10
	L161	S3		32.4	11.3	0.11
	D164			42.6	3.5	0.06
	D165			30.0	21.3	-0.27
	F168			63.7	6.8	0.11
	F189	S4		34.2	0.3	0.00
	W193			41.1	19.8	0.30
	L350	S6		18.6	0.5	0.01
	L353		H	53.8	17.7	0.22
	W354			24.9	22.1	0.17
	T356			41.9	0.7	0.01
	T357			27.6	16.2	0.11
	H358			2.8	1.4	0.02
#2 [1]	D99	S2	H	65.1	10.4	-0.04
	V102			16.4	15.4	0.25
	V103			26.0	0.7	0.01
	L161	S3		31.1	9.1	0.13
	D165			40.4	25.1	-0.37
	F168			68.7	9.5	0.15
	F189	S4		45.4	20.3	0.32
	W193			15.7	10.1	0.11
	L350	S6		18.6	0.3	0.01
	L353			50.3	11.1	0.13
	W354			23.8	22.1	0.20
	T357			13.0	9.4	0.11
	H358			2.5	1.6	0.02
#3 [1]	D99	S2		60.5	8.4	-0.03
	V102			19.9	19.3	0.31
	V103			24.0	0.3	0.01
	L161	S3		32.3	11.3	0.18
	L162			11.1	0.8	0.01
	D165		H	44.0	35.1	-0.47
	F168			67.5	9.7	0.15
	F189	S4		42.9	16.9	0.27
	W193			23.2	16.8	0.23
	L350	S6		18.6	0.5	0.01
	L353			47.8	9.7	0.10
	W354			25.1	23.4	0.19
	T357			13.1	10.1	0.11
	H358			2.5	1.6	0.02
#4 [1]	D99	S2		61.6	10.6	-0.04
	V102			22.6	16.3	0.26
	V103			22.7	0.8	0.01
	L161	S3		32.9	9.0	0.14
	D165			30.1	20.7	-0.31
	F168			65.9	8.7	0.14
	F189	S4		30.4	6.1	0.10
	W193			39.9	22.4	0.30
	L353	S6		49.5	10.0	0.11
	W354			24.4	22.6	0.19
	T357			33.8	18.0	0.23
	H358			3.0	1.6	0.02
#5 [2]	D165	S3		24.5	7.3	0.01
	F166			41.5	11.0	0.18
	F168			65.3	20.4	0.32
	Y169	Loop		83.5	19.7	0.31
	A170		H	51.8	14.9	0.11
	K175			108.7	9.2	0.12
	E181	S4		100.6	6.3	0.10
	I182			51.0	10.4	0.17
	N185		H	76.6	35.5	-0.32
	F189			36.0	4.3	0.07
#6 [2]	D165	S3		25.1	7.1	0.01
	F166			41.5	10.9	0.17
	F168			65.3	17.8	0.29
	Y169	Loop		83.5	19.8	0.32
	A170			51.8	11.6	0.06
	M171			152.5	0.2	0.00
	K175			108.7	14.8	0.19
	E181	S4		100.6	14.3	0.23
	I182			51.0	11.2	0.18
	N185		H	75.3	39.0	-0.34
	F189			33.0	4.1	0.06
#7 [2]	D165	S3		24.8	11.9	-0.04
	F166			42.6	12.1	0.19
	F168			67.0	21.4	0.33
	Y169	Loop		83.5	19.7	0.31
	A170			51.8	14.7	0.11
	K175			108.7	8.7	0.11
	E181	S4		100.6	5.8	0.09
	I182			51.0	9.9	0.16
	N185		H	82.7	40.0	-0.35
	F189			24.6	2.8	0.04
#8 [2]	D165	S3	H	24.5	11.5	-0.05
	F166			42.6	11.4	0.18
	F168			67.1	18.0	0.29
	Y169	Loop		83.5	19.8	0.32
	A170			51.8	11.6	0.06
	M171			152.5	0.2	0.00
	K175			108.7	14.8	0.19
	E181	S4		100.6	14.5	0.23
	I182			51.0	11.2	0.18
	N185		H	83.0	43.8	-0.39
#9 [3]	C68	S1		60.4	35.6	0.36
	T69			30.1	6.7	0.11
	G72			25.5	10.1	0.16
	D99	S2		55.6	1.6	0.02
	I100			38.3	6.0	0.10
	V103			27.7	19.5	0.31
	N185	S4		77.5	27.1	-0.13
	G188			13.1	4.9	0.08
	F189		H	24.0	10.9	0.17
	L192			10.4	8.5	0.14
#10 [3]	C68	S1		60.4	35.8	0.36
	T69		H	30.1	6.9	0.11
	G72			25.5	10.4	0.17
	D99	S2		69.5	2.4	0.04
	I100			38.3	6.2	0.10
	V103			30.0	20.2	0.32
	N185	S4		86.8	27.3	-0.13
	G188			13.0	5.0	0.08
	F189			32.1	9.7	0.15
	L192			10.2	8.2	0.13

Table S1. β-D-Fructofuranose binding residues in each binding mode. Residues located at the interface between the BmGr9 model and β-D-fructofuranose of 10 docking modes were analyzed using PDBePISA. Res., residue; Bond, the existence of hydrogen bonds; ASA (Accessible Surface Area), area of a monomeric unit; residue, or atom, which is accessible to solvent; BSA (Buried Surface Area), surface area buried in the interface for each monomer; ΔG^i (solvation energy effect), energy difference between bound and unbound states of monomeric units, residues, or atoms, which is due to the solvation effect.
